# Diabetic pregnancy as a novel risk factor for cardiac dysfunction in the offspring—the heart as a target for fetal programming in rats

**DOI:** 10.1007/s00125-021-05566-5

**Published:** 2021-09-18

**Authors:** Till Schütte, Sarah M. Kedziora, Nadine Haase, Florian Herse, Natalia Alenina, Dominik N. Müller, Michael Bader, Michael Schupp, Ralf Dechend, Michaela Golic, Kristin Kräker

**Affiliations:** 1 Charité - Universitätsmedizin Berlin, corporate member of Freie Universität Berlin and Humboldt-Universität zu Berlin, Berlin, Germany; 2 Charité - Universitätsmedizin Berlin, corporate member of Freie Universität Berlin and Humboldt-Universität zu Berlin, Institute of Pharmacology, Berlin, Germany; 3 Berlin Institute of Health at Charité - Universitätsmedizin Berlin, Berlin, Germany; 4 DZHK (German Center for Cardiovascular Research), Partner Site Berlin, Berlin, Germany; 5 Max Delbrück Center for Molecular Medicine in the Helmholtz Association, Berlin, Germany; 6 Experimental and Clinical Research Center – a joint cooperation between the Max Delbrück Center for Molecular Medicine and the Charité – Universitätsmedizin Berlin, Berlin, Germany; 7 Institute for Biology, University of Lübeck, Lübeck, Germany; 8 HELIOS-Klinikum, Department of Cardiology and Nephrology, Berlin, Germany; 9 HSD Hochschule Döpfer, University of Applied Sciences, Cologne, Germany

**Keywords:** Cardiovascular diseases, Diabetes mellitus, Diet, Echocardiography, High-fat, Hyperglycaemia, Infant, Maternal inheritance, Pregnancy, Rats, Transgenic

## Abstract

**Aims/hypothesis:**

The impact of diabetic pregnancy has been investigated extensively regarding offspring metabolism; however, little is known about the influence on the heart. We aimed to characterise the effects of a diabetic pregnancy on male adult offspring cardiac health after feeding a high-fat diet in an established transgenic rat model.

**Methods:**

We applied our rat model for maternal type 2 diabetes characterised by maternal insulin resistance with hyperglycaemia and hyperinsulinaemia. Diabetes was induced preconceptionally via doxycycline-induced knock down of the insulin receptor in transgenic rats. Male wild-type offspring of diabetic and normoglycaemic pregnancies were raised by foster mothers, followed up into adulthood and subgroups were challenged by a high-fat diet. Cardiac phenotype was assessed by innovative speckle tracking echocardiography, circulating factors, immunohistochemistry and gene expression in the heart.

**Results:**

When feeding normal chow, we did not observe differences in cardiac function, gene expression and plasma brain natriuretic peptide between adult diabetic or normoglycaemic offspring. Interestingly, when being fed a high-fat diet, adult offspring of diabetic pregnancy demonstrated decreased global longitudinal (−14.82 ± 0.59 vs −16.60 ± 0.48%) and circumferential strain (−23.40 ± 0.57 vs −26.74 ± 0.34%), increased relative wall thickness (0.53 ± 0.06 vs 0.37 ± 0.02), altered cardiac gene expression, enlarged cardiomyocytes (106.60 ± 4.14 vs 87.94 ± 1.67 μm), an accumulation of immune cells in the heart (10.27 ± 0.30 vs 6.48 ± 0.48 per fov) and higher plasma brain natriuretic peptide levels (0.50 ± 0.12 vs 0.12 ± 0.03 ng/ml) compared with normoglycaemic offspring on a high-fat diet. Blood pressure, urinary albumin, blood glucose and body weight were unaltered between groups on a high-fat diet.

**Conclusions/interpretation:**

Diabetic pregnancy in rats induces cardiac dysfunction, left ventricular hypertrophy and altered proinflammatory status in adult offspring only after a high-fat diet. A diabetic pregnancy itself was not sufficient to impair myocardial function and gene expression in male offspring later in life. This suggests that a postnatal high-fat diet is important for the development of cardiac dysfunction in rat offspring after diabetic pregnancy. Our data provide evidence that a diabetic pregnancy is a novel cardiac risk factor that becomes relevant when other challenges, such as a high-fat diet, are present.

**Graphical abstract:**

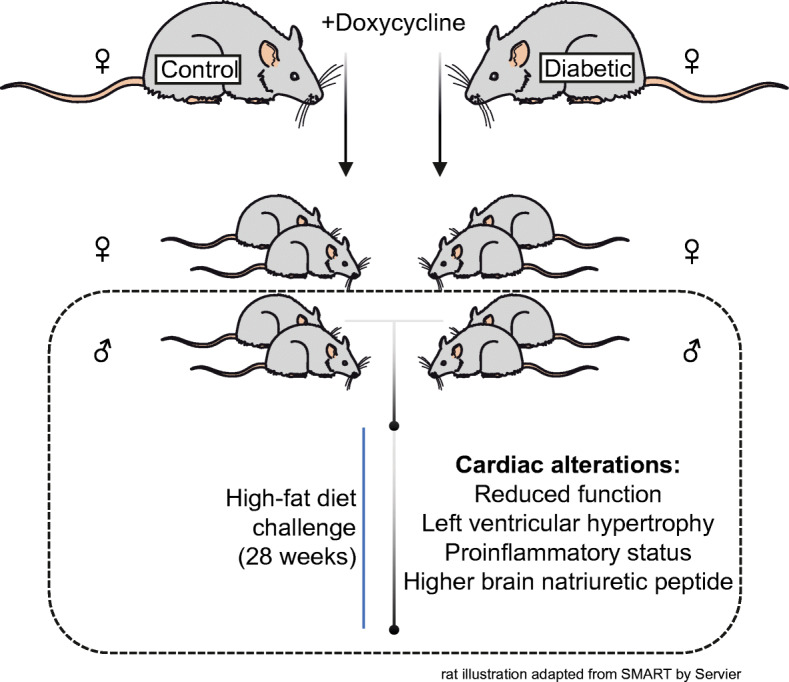

**Supplementary Information:**

The online version of this article (10.1007/s00125-021-05566-5) contains peer-reviewed but unedited supplementary material.

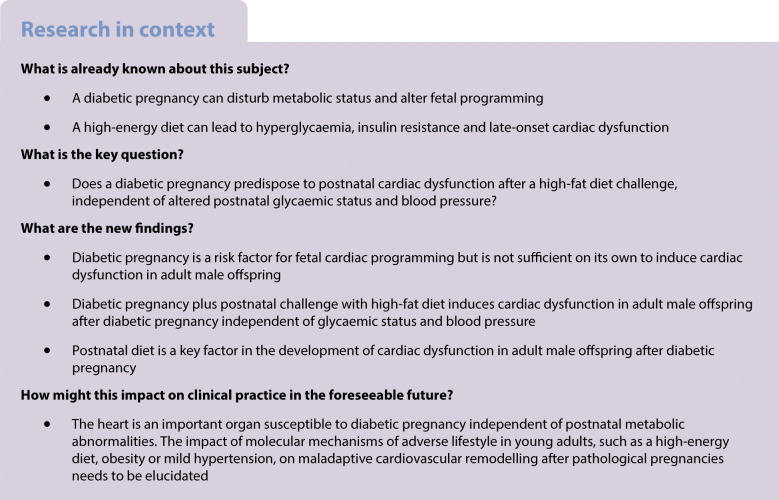



## Introduction

Diabetes is a global pandemic with an increasing prevalence; almost 500 million people have diabetes today [1]. Rising prevalence is also seen in young people [2], as well as pregnant women who may have type 1 or type 2 diabetes preconceptionally or develop gestational diabetes, defined as the first manifestation of diabetes during pregnancy. Gestational diabetes is one of the most widespread complications of pregnancy, with a prevalence up to 10% of all pregnant women in the USA [3].

Several studies have investigated the influence of maternal hyperglycaemia on offspring health, based on the concept of fetal programming. It has been suggested that the effect of the intrauterine environment on later offspring health may be modulated by postnatal environmental factors such as growth [4] and nutrition. Most data on the influence of diabetes during pregnancy focuses on the metabolic status of the offspring such as obesity [5–9] and diabetes [6, 8] and there is much less information about the impact on cardiac function. Adverse impacts during the prenatal period may programme an elevated risk for cardiovascular outcome in later life, but underlying mechanisms are still inadequately understood, although evidence is slowly emerging on the influence of BP [10].

A recent cohort study from Denmark showed that children and adults aged under 40 years have a higher risk for early onset CVD if their mothers had diabetes before or during pregnancy [11]. BP of children whose mothers were diabetic during pregnancy is higher than BP of control children [12–14]. Interestingly, male offspring seem more vulnerable to higher BP after a pregnancy in which the mother had diabetes [13, 14]. Cardiac dysfunction and gene expression alteration was reported in fetuses of pregestational hyperglycaemic rats [15]. Hyperglycaemic perfusion of rat uterus provokes septal overgrowth in fetal hearts [16]. Interestingly, maladaptive cardiac gene expression profiles induced by maternal hyperglycaemia turn transiently normal in rat neonates [17].

Mechanisms that may induce cardiac remodelling and dysfunction are scarcely investigated, but in rodent models of heart failure, dietary fat intake often plays an important role in attenuation or prevention of cardiac dysfunction [18]. Obesity-prone rats on a moderate-fat diet did not exhibit hypertension and cardiac dysfunction [19]. By contrast, a high-fat diet (HFD) is able to promote cardiac hypertrophy in rats [20]. However, few studies have sufficiently examined the outcome of a diabetic pregnancy combined with a dietary challenge in later life of the progeny, a realistic scenario in humans, and examined the heart as a primary target of investigation.

Therefore, we aimed to characterise the cardiac phenotype of HFD-fed adult male offspring descended from a diabetic dam in a transgenic rat model [21–23] based on insulin receptor knockdown mediated by small hairpin RNA (shRNA). We performed an extensive, thorough study on offspring health in this model and have already shown that there is no difference in glucose intolerance and body weight in adult male offspring both on normal chow (NC) and HFD after a diabetic pregnancy compared with a control pregnancy [23]. This gives us the opportunity to investigate potential cardiac maladaptation independent of metabolic risk factors. We report here the results of the cardiac phenotyping in this study. We applied innovative methods such as speckle tracking echocardiography (STE) and focused on male offspring considered to be more vulnerable to cardiovascular programming [13, 14]. We hypothesised that in our transgenic rat model, a diabetic pregnancy impairs cardiac function in the adult offspring when fed an HFD.

## Methods

### Animal model

The selected transgenic Tet29 rat model [21] was generated on a Sprague-Dawley background and carries a DNA construct producing a shRNA that interferes with insulin receptor expression. The resulting insulin receptor knock down is inducible by doxycycline (DOX) and generates hyperglycaemia and hyperinsulinaemia. The mechanism is tightly regulated; when DOX treatment is stopped the insulin receptor knock down will diminish. Adequate animal housing was carried out under standard conditions with an mean room temperature of 22°C, a humidity of 55 ± 15% and 12/12 h light/dark cycle. Rats had ad libitum access to food (NC: 54% carbohydrates, 36% protein, 10% fat, V1324-300, ssniff Spezialdiäten, Soest, Germany; HFD: 35% carbohydrates, 20% protein, 45% fat, D12451i, Research Diets, New Brunswick, USA). After completion of experiments, rats were decapitated under 1.5% isoflurane anaesthesia. All experimental animal protocols were approved by local authorities (State Office of Health and Social Affairs, Berlin, Germany). Moreover, we adhere to the European law for animal protection with respect to the National Research Council (NRC) Guide [24].

### Experimental design

**Fig. 1:**
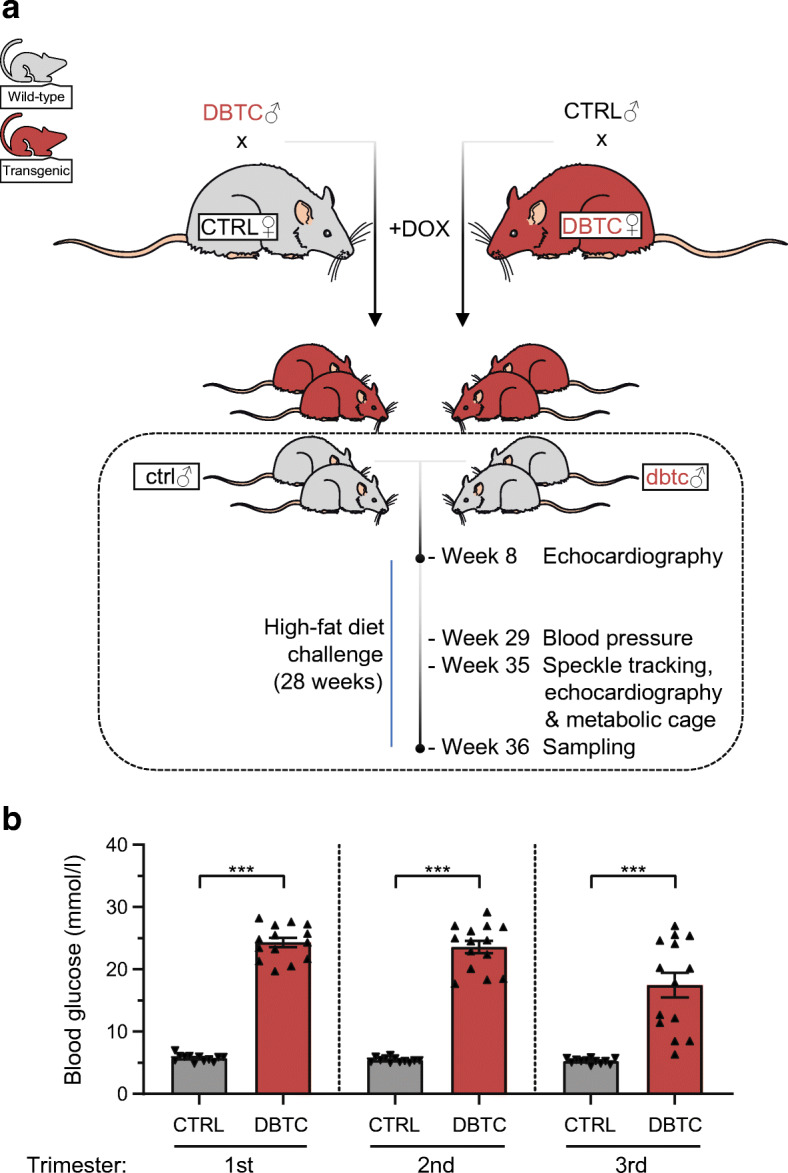
Experimental mating scheme and follow-up of transgenic rat model and maternal blood glucose during pregnancy. Parental WT females (CTRL) and transgenic Tet29 (TG) females (DBTC) were administered 1.5 mg/kg DOX via drinking water preconceptionally. WT females were mated with TG males (DBTC) and TG females with WT males (CTRL). DOX treatment was stopped once a vaginal plug was registered. Male WT offspring of normoglycaemic (ctrl) and diabetic (dbtc) dams were followed up (dashed box). Echocardiography was conducted at the age of 8 weeks prior to dietary challenge lasting 28 weeks. BP measurements were performed at the age of 29 weeks. Echocardiography, in-depth speckle tracking analysis and metabolic cage were assessed at the age of 35 weeks. At the end of the challenge (week 36 of age) samples were extracted (**a**). TG females have significantly higher mean blood glucose levels accumulated during each trimester of pregnancy (**b**). Normoglycaemic (CTRL, *n*=12) and diabetic dams (DBTC, *n*=14), mean±SEM, unpaired Student’s *t* test (**b**), ***p≤0.001. This figure was created using Servier Medical Art (https://smart.servier.com/). Servier Medical Art by Servier is licensed under a Creative Commons Attribution 3.0 Unported License

To induce a diabetic milieu during pregnancy, transgenic Tet29 female dams (*n* = 14) ingested 1.5 mg/kg body weight DOX orally by drinking water. Mating to wild-type (WT) males was initiated when treated Tet29 females hit a hyperglycaemic blood glucose level of about 16.65 mmol/l. To prevent embryotoxicity/fetotoxicity, DOX administration was stopped when females showed a vaginal plug (defined as pregnancy day 1). It took 9.8 days of DOX administration on mean until conception, but the DOX effect on the blood glucose level persisted throughout pregnancy (Fig. 1b). Female WT dams (*n* = 12) were mated with transgenic Tet29 males and treated with 1.5 mg/kg body weight DOX as well. Here, mean DOX administration prior to conception was 9.3 days. At the end of pregnancy, blood samples were collected from dams for further analysis. WT nursing dams were bred alongside to facilitate DOX-free breastfeeding. The offspring delivery required Caesarean section on pregnancy day 22, and pups were raised by nursing dams to avoid the bias of being suckled by hyperglycaemic dams. Pups were followed up to adulthood and finally challenged with an HFD. For this purpose, groups were allocated in a randomised fashion to avoid litter effects. Hence, four groups were composed from normo- or hyperglycaemic pregnancy being fed either NC (*n* = 8,8) or HFD (*n* = 15,10). At week 36 of age, offspring were decapitated, weighed, blood was collected and organs were withdrawn, weighed and cryogenically preserved. The metabolic characterisation of the animals within this study including GTT, ITT and telemetric continuous glucose measurement has already been reported [23]. We focus here on the cardiac analysis of the animals.

### BP measurement

Accurate BP measurement was performed by non-invasive tail cuff technique (CODA High Throughput System, Kent Scientific Corp., Torrington, USA). The volume pressure recording sensor technology provides systolic, diastolic and mean arterial BP (MAP) and heart rate.

### Blood glucose metering

Glucose metering was determined by taking advantage of the Accu-Chek Aviva blood glucose meter (Roche, Mannheim, Germany). Capillary blood droplets were drawn by puncture of the tail.

### Circulatory and urinary factors

Circulating brain natriuretic peptide (BNP) was measured in blood plasma samples by an ELISA kit according to manufacturer’s instructions (BNP45, Abcam, Cambridge, UK). Urinary albumin was quantified by the Core Facility Animal Phenotyping of the Max Delbrück Center for Molecular Medicine in the Helmholtz Association.

### Echocardiography and advanced in-depth speckle tracking

Transthoracic echocardiography was performed in anaesthetised (1.5% isoflurane) animals. ECG, respiration and temperature were constantly controlled during the procedure. A Vevo 3100 high-resolution imaging system (Fujifilm VisualSonics, Inc., Toronto, Canada) was used and paired with a 21-MHz transducer (MS250) mounted on an integrated rail system. Imaging data was captured for in silico as well as blinded observer analysis using VevoStrain software (version 2.2.0; Fujifilm VisualSonics, Inc., Toronto, Canada). B-mode cine loops were used in parasternal long- and short-axis view to assess basic variables for systolic function and advanced in-depth STE analysis. Imaging data was peer-reviewed for quality regarding differentiation of wall borders and general absence of artefacts. In-depth analysis was performed over three sequential cardiac cycles and means were calculated. M-mode was used to measure cardiac wall and chamber dimensions. Relative wall thickness (RWT) was calculated by dividing the double posterior wall thickness by the inner diameter of the left ventricle in the end-diastolic state. Body weight was recorded during this procedure.

### mRNA isolation and quantitative real-time PCR

Pieces of frozen cardiac tissue were homogenised using ceramic beads. Total RNA was isolated using commercial kits (QIAzol lysis reagent and RNeasy Mini Kit, Qiagen, Hilden, Germany) according to protocols provided by the manufacturer. A total of 2 μg of RNA was transcribed into cDNA by High Capacity cDNA Reverse Transcription Kit (Applied Biosystems, Vilnius, Lithuania). Relative quantification of gene expression was performed by quantitative real-time PCR (qPCR) using an ABI 7500 Fast Sequence Detection System (Applied Biosystems, Foster City, USA). Primers and probes (ESM Table 1) were designed with Primer Express 3.0 (Applied Biosystems, Foster City, USA), pre-validated by in silico blasting and synthesised by Biotez (Berlin, Germany). Quantitative analysis of target mRNA expression was performed with qPCR using the relative standard curve method. qPCR was normalised to the *36B4* gene (also known as *Rplp0*).

### Immunohistochemistry

After formalin fixation and paraffin embedding, hearts were cut through the short axis in 2 μm sections. Samples (*n* = 6 per group) were stained for wheat germ agglutinin (WGA; No. FL-1021; Vector Laboratories, Burlingame, USA), collagen type 1 (COL1; No. 1310-01; SouthernBiotech, Birmingham, USA) and macrosialin (ED1; No. MCA341R; Bio-Rad Laboratories, Feldkirchen, Germany). Nuclei were stained by Vectashield mounting medium with DAPI (No. H-1200; Vector Laboratories). Slides were imaged by Panoramic MIDI II (3DHISTECH, Budapest, Hungary) and evaluated using associated analysis software (Case Viewer, 3DHISTECH). Perimeters of 20 randomly selected cardiomyocytes per section were framed manually (WGA staining). Perivascular fibrosis was assessed with regard to internal vessel diameter, vessel wall and fibrotic border (COL1 staining). Inflammatory status was defined by manually counted CD68-positive cells in five representative microscopic fields of view (fov, ×40; ED1 staining). A blind observer calculated mean scores of each animal to deduce a group mean score. To ensure that the secondary antibody binds specifically, negative controls were used without primary antibody.

### Metabolic cage

Animals were separated over 24 h to collect accumulative urine in metabolic cages (Tecniplast, Gazzada, Italy) under standard conditions. Urinary samples were centrifuged to remove solid components. The supernatant was stored at −20°C.

### Statistical analysis

Statistical analyses were performed by Prism 8.0 software (GraphPad Software, La Jolla, USA). The ROUT method was chosen for outlier identification with an average false discovery rate (Q) of <1%. After testing for gaussian distribution, group differences were analysed by an unpaired test (parametric Student’s *t* test or nonparametric Mann–Whitney *U* test). Data are presented as mean ± SEM and two-sided *p* ≤ 0.05 was considered statistically significant.

## Results

The study design is presented in Fig. 1a. Female transgenic rats (DBTC) were crossed with WT males (CTRL) after administration of DOX. Non-transgenic male offspring of diabetic (dbtc) and normoglycaemic pregnancy (ctrl) were used for further studies up to an age of 36 weeks (Fig. 1a). During pregnancy, diabetic DBTC dams showed significantly higher blood glucose levels in contrast to normoglycaemic CTRL dams in each trimester with 24.34 ± 0.74 vs 5.75 ± 0.16 mmol/l in the first, 23.60 ± 0.99 vs 5.44 ± 0.11 mmol/l in the second and 17.48 ± 1.96 vs 5.26 ± 0.12 mmol/l in the third trimester (Fig. 1b). Maternal blood glucose is based on data from Schütte et al and was appropriately adapted [23]. Litter sizes were not altered between diabetic and control pregnancies, but mean birthweight of all pups was decreased in diabetic pregnancies compared with control pregnancies (data not shown). However, only the heavier diabetic pregnancy offspring which did not differ in body weight from normoglycaemic pregnancy offspring survived the raising by foster mothers and could be taken into investigation and analysis of our study [23].

**Fig. 2:**
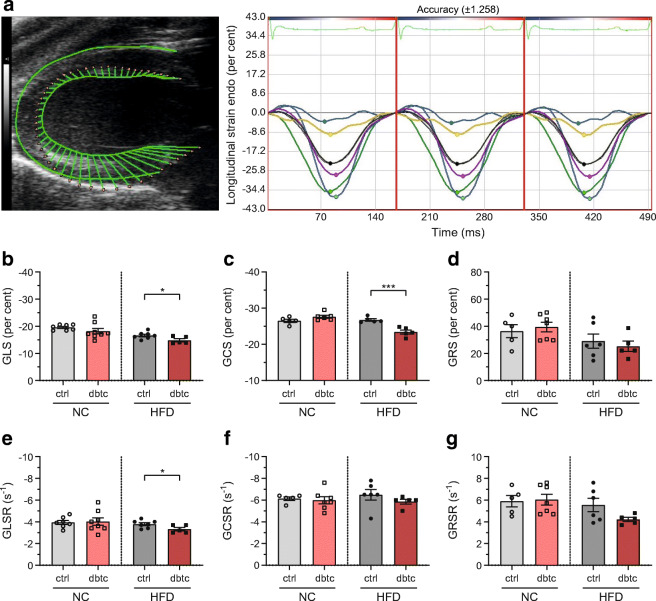
In-depth analysis of offspring by STE after dietary challenge. Tissue dynamics of the left ventricle was measured in multiple single dots mapping the endocardium. Segmental values are summarised in six defined regions (green: posterior base, white: posterior middle (partially obscured by black line), cyan: posterior apex, blue: anterior base, yellow: anterior middle, pink: anterior apex, black: average), global values are calculated in means over three cardiac cycles (VevoStrain software, version 2.2.0) (**a**). GLS, GCS and GRS (**b**–**d**) as well as their rates (GLSR, GCSR, GRSR) (**e**–**g**) were unaltered in NC-fed dbtc offspring compared with ctrl. When fed an HFD, GLS and GCS (**b**, **c**) were significantly reduced in dbtc offspring compared with ctrl, whereas GRS (**d**) was unaltered. GLSR was reduced after HFD (**e**). Residual circumferential and radial strain rates (**f**, **g**) were not reduced after HFD. Male WT offspring of normoglycaemic (ctrl, NC; **b**, **e** *n*=7; **c**, **d**, **f**, **g** *n*=5; HFD; **b**, **e** *n*=7; **c**, *n*=5; **d**, **f**, **g** *n*=6) and diabetic dams (dbtc, NC; **b**, **e** *n*=8; **c**, *n*=6; **d**, **f**, **g** *n*=7; HFD; **b**–**f**, *n*=5), GLS/R was assessed from longitudinal axis and GCS/R and GRS/R from short axis, echocardiography at 35 weeks of age, unpaired Student’s *t* test (**b**, **c**, **d**, **e**, **g**) and Mann–Whitney *U* test (**f**), mean±SEM, *p≤0.05, ***p≤0.001

Next, we analysed the cardiovascular end organ damage in the offspring at the end of the dietary challenge, which is shown in Fig. 2. In-depth STE analysis was performed to characterise cardiac function (Fig. 2a). When being fed NC, no noteworthy differences were found in dbtc offspring compared with ctrl among all strains and affiliated rates (Fig. 2b–g). However, after an HFD challenge, STE demonstrated that global longitudinal strain (GLS) and global circumferential strain (GCS) were significantly reduced in dbtc offspring compared with ctrl, with −14.82 ± 0.59 vs −16.60 ± 0.48% and –23.40 ± 0.57 vs −26.74 ± 0.34% (Fig. 2b,c). Global radial strain (GRS) of dbtc offspring compared with ctrl was unaltered, with 25.22 ± 3.82 vs 29.07 ± 5.23% (Fig. 2d). In accordance with the significant GLS reduction, its rate (GLSR) was significantly reduced with −3.32 ± 0.16 vs −3.79 ± 0.13 s^−1^ comparing offspring with diabetic and normoglycaemic pregnancy origin (Fig. 2e). The GCS rate (GCSR) showed no reduction with −5.86 ± 0.22 vs −6.48 ± 0.49 s^−1^ in dbtc offspring compared with ctrl (*p* = 0.06) (Fig. 2f). GRS rate (GRSR) of dbtc offspring compared with ctrl displayed no reduction with 4.22 ± 0.19 vs 5.55 ± 0.62 s^−1^ (*p* = 0.09) (Fig. 2g).

**Fig. 3:**
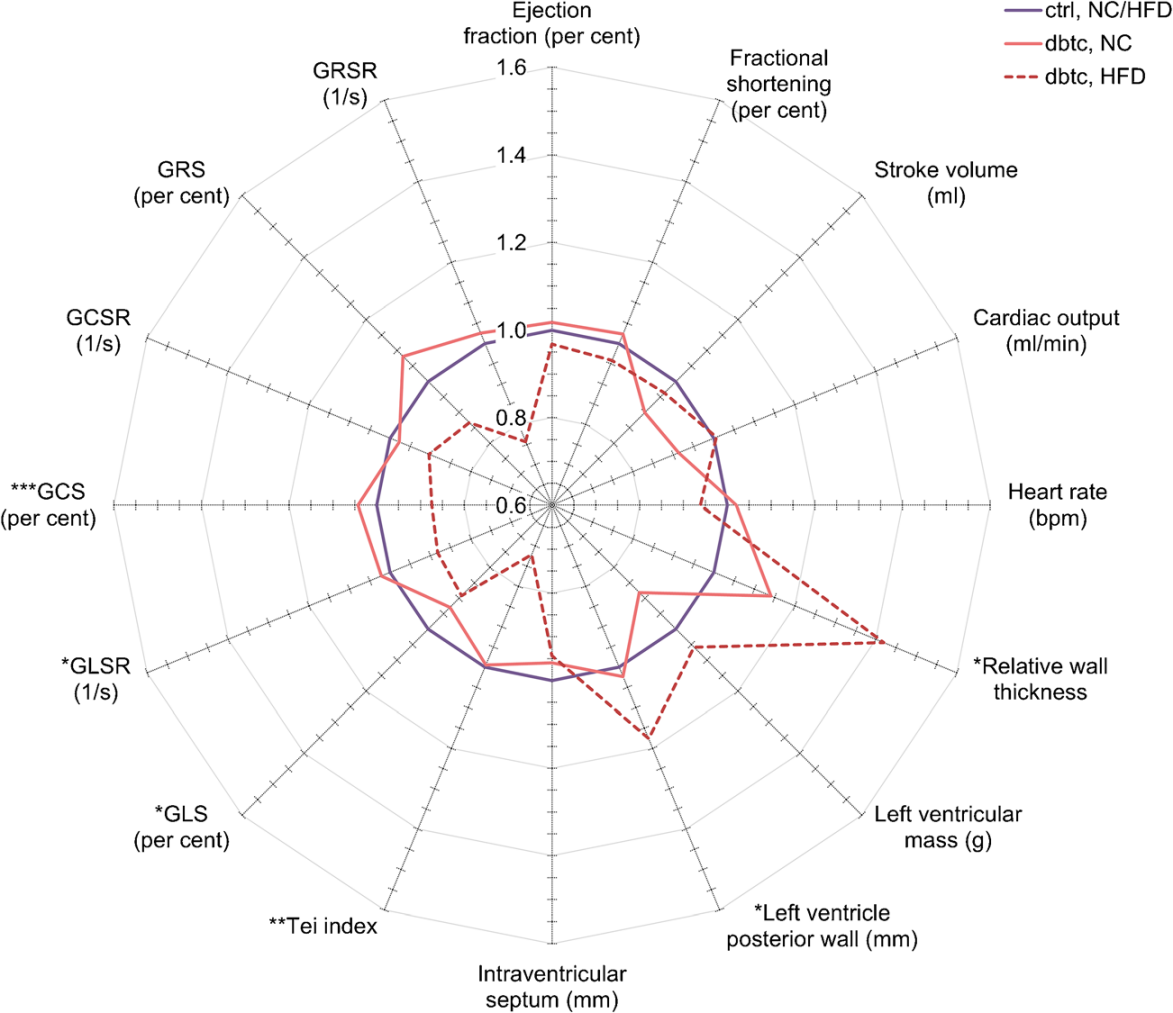
Aggregated basic echocardiography and in-depth analysis of dietary challenged offspring. The data of male WT offspring of normoglycaemic dams normalised to 1 (purple line, NC/HFD, *n*=7/7) and then merged. Male WT offspring of diabetic pregnancy are compared with male WT offspring of control pregnancy independent of food (NC or HFD). Relative alterations in male WT diabetic offspring fed NC (solid red line, *n*=8) or HFD (dotted red line, *n*=5) are shown. Echocardiography was assessed at 35 weeks of age. Relative mean values, unpaired Student’s *t* test and Mann–Whitney *U* test, *p≤0.05, **p≤0.01, ***p≤0.001

To visualise the cardiac dysfunction, we showed the echocardiography analysis as a spider blot in (Fig. 3). The purple line (ctrl, NC/HFD) represents the ctrl offspring normalised to 1, whereas the solid red line (dbtc, NC) and the dotted red line (dbtc, HFD) represent altered dbtc offspring in relation to offspring from normoglycaemic pregnancies. When being fed NC, no significant differences were present in dbtc compared with ctrl. The most prominent alterations took place after HFD challenge with a reduction in GLS, GLSR and GCS in offspring of diabetic pregnancy. Additionally, the Tei index, an established myocardial performance index (MPI) and a marker to identify cardiac dysfunction, was significantly reduced in dbtc compared with ctrl offspring, with 0.98 ± 0.06 vs 1.36 ± 0.07. Interestingly, we also observed evidence of cardiac hypertrophy since the left ventricular posterior wall and the RWT were significantly increased in dbtc offspring fed an HFD with 2.16 ± 0.11 vs 1.84 ± 0.04 mm and 0.53 ± 0.06 vs 0.37 ± 0.02 compared with ctrl.

Absolute values of echocardiography after NC and HFD are listed in Table 1. Echocardiography at the age of 8 weeks performed prior to the dietary challenge showed no significant alterations between dbtc and ctrl offspring (ESM Table 2).

**Table 1:** Basic echocardiography and in-depth analysis of dietary challenged offspring

Basic echocardiography and in-depth speckle tracking analysis	NC			HFD		
	ctrl	dbtc	*p* value	ctrl	dbtc	*p* value
	Mean±SEM	Mean±SEM		Mean±SEM	Mean±SEM	
Conventional variable						
Ejection fraction (per cent)	59.17 ± 1.80	60.22 ± 2.28	0.7285	60.52 ± 2.39	58.58 ± 2.89	0.6163
Fractional shortening (per cent)	32.84 ± 1.11	33.62 ± 1.40	0.6743	35.51 ± 1.44	34.02 ± 1.97	0.5440
Stroke volume (ml)	427.92 ± 13.60	385.01 ± 23.52	0.1527	411.89 ± 37.43	396.76 ± 32.17	0.7780
Cardiac output (ml/min)	129.07 ± 6.71	117.78 ± 6.71	0.2576	123.60 ± 4.45	124.38 ± 18.73	0.9652
Heart rate (bpm)	302.14 ± 13.97	308.38 ± 13.39	0.6343	328.00 ± 10.39	308.00 ± 24.37	0.4188
Extended morphology						
RWT	0.36 ± 0.03	0.41 ± 0.04	0.3757	0.37 ± 0.02	0.53 ± 0.06	0.0200
Left ventricular mass (g)	1473.88 ± 34.82	1298.96 ± 83.62	0.1120	1349.23 ± 46.36	1427.41 ± 99.22	0.4490
Left ventricle posterior wall (mm)	1.83 ± 0.06	1.88 ± 0.10	0.7512	1.84 ± 0.04	2.16 ± 0.11	0.0117
Intraventricular septum (mm)	1.83 ± 0.04	1.76 ± 0.07	0.6620	1.70 ± 0.07	1.60 ± 0.13	0.4906
Myocardial performance						
Tei index	1.13 ± 0.09	1.12 ± 0.09	0.9726	1.36 ± 0.07	0.98 ± 0.06	0.0040
GLS (per cent)	−19.57 ± 0.38	−18.20 ± 1.03	0.2571	−16.60 ± 0.48	−14.82 ± 0.59	0.0403
GLSR (1/s)	−3.94 ± 0.19	−4.03 ± 0.34	0.8435	−3.79 ± 0.13	−3.32 ± 0.16	0.0487
GCS (per cent)	−26.50 ± 0.45	−27.63 ± 0.42	0.0968	−26.74 ± 0.34	−23.40 ± 0.57	0.0010
GCSR (1/s)	−6.14 ± 0.17	−5.99 ± 0.34	0.4987	−6.48 ± 0.49	−5.86 ± 0.22	0.0649
GRS (per cent)	36.44 ± 4.78	39.40 ± 3.52	0.6201	29.07 ± 5.23	25.22 ± 3.82	0.5819
GRSR (1/s)	5.90 ± 0.53	6.04 ± 0.50	0.8513	5.55 ± 0.62	4.22 ± 0.19	0.0939

Conventional echocardiography, extended morphology and myocardial performance variables assembled from basic echocardiography and speckle tracking analysis. Male WT offspring of normoglycaemic (ctrl, NC/HFD, *n*=7/7) and diabetic dams (dbtc, NC/HFD, *n*=8/5), echocardiography assessed at 35 weeks of age, unpaired Student’s *t* test and Mann–Whitney *U* test, mean±SEM, *p* values are given

**Fig. 4:**
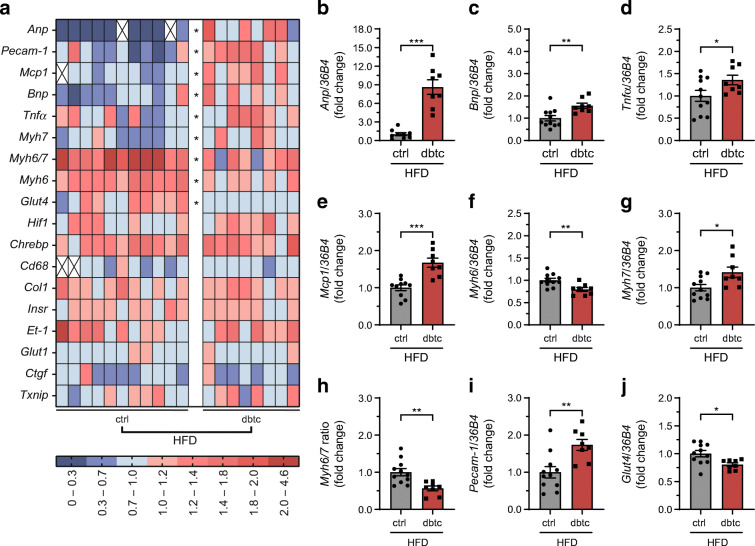
Cardiac gene expression profile of HFD challenged offspring shown as heatmap and selected genes. Gene expression profile of the heart apex after HFD challenge acquired by qPCR and illustrated as a heatmap (expression visualised by colour scale) showing absolute values (**a**) and selected genes (**b**–**j**) in reference to *36B4* housekeeping gene expression. Dbtc offspring exhibited a significantly different gene expression profile compared with ctrl. Offspring of normoglycaemic (ctrl, *n*=11) and diabetic dams (dbtc, *n*=8), qPCR performed at 36 weeks of age, unpaired Student’s *t* test (**b**, **c**, **e**, **f**, **g**, **h**, **i**, **j**) and Mann–Whitney *U* test (**d**), single values (**a**), outlier marked with X (**a**), normalised mean±SEM (**b**–**j**), *p≤0.05, **p≤0.01, ***p≤0.001

To investigate if the functional differences are accompanied by structural alterations, we performed qPCR analysis of marker genes for cardiac end organ damage. The gene expression profile after HFD challenge is demonstrated as a consolidated heatmap and altered genes are highlighted in Fig. 4. Dbtc offspring exhibited a significantly different gene expression profile compared with ctrl. The most striking result of the analysis was a significant increase of atrial natriuretic peptide (*Anp*, also known as *Nppa*, Fig. 4b) and BNP (*Bnp*, also known as *Nppb*, Fig. 4c). Interestingly, *Tnfα*, (also known as *Tnf*, Fig. 4d) and monocyte chemoattractant protein (*Mcp1*, also known as *Ccl2*, Fig. 4e) were significantly increased as well. Importantly, the significant decrease of myosin heavy chain α (*Myh6*, Fig. 4f) paralleled by the significant increase in myosin heavy chain β (*Myh7*, Fig. 4g) resulted in a significantly decreased *Myh6*/*Myh7* ratio (Fig. 4h) providing strong evidence for myocardial dysfunction (Fig. 4f–h). In addition, platelet endothelial cell adhesion molecule (*Pecam-1*, Fig. 4i) was significantly increased and *Glut4* (also known as *Slc2a4*, Fig. 4j) decreased.

Similar to the functional data, no significant alterations were observed in the cardiac gene expression profile when feeding NC to dbtc in comparison with ctrl offspring (ESM Fig. 1). Absolute values of gene expression analysis in all groups are given in ESM Table 3.

**Fig. 5:**
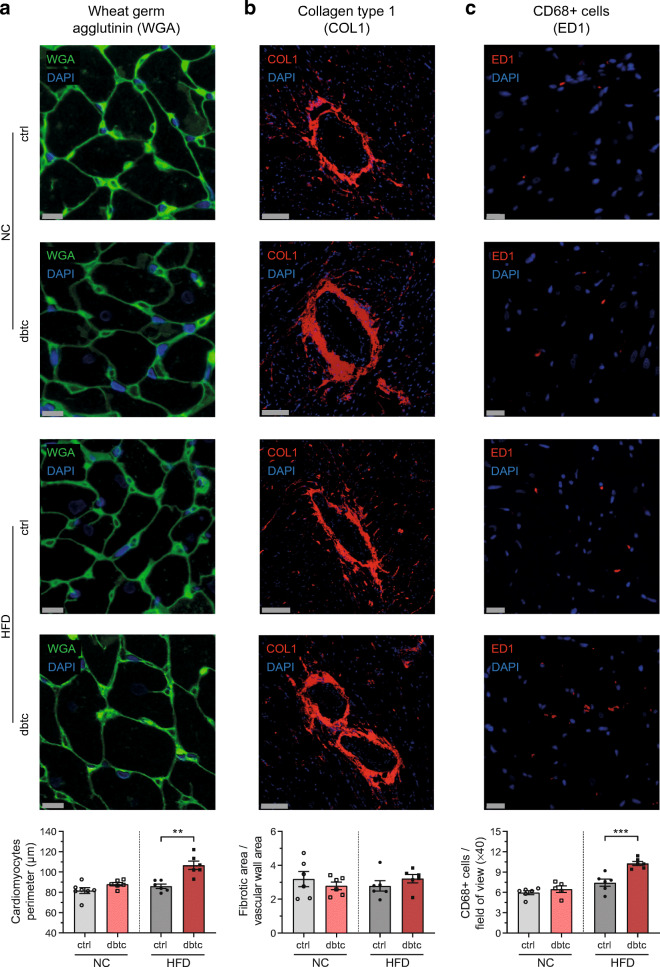
Immunohistochemistry of cardiac tissue in offspring. Cardiac cross-sections were stained with WGA to measure perimeters of cardiomyocytes, which were significantly increased in HFD-fed dbtc offspring compared with ctrl (**a**). COL1 was measured to compare area of perivascular fibrosis in relation to vessel wall area. It was unaltered in dbtc offspring compared with ctrl (**b**). ED1, detecting number of CD68-positive cells, was significantly increased in dbtc offspring compared with ctrl (**c**). Male WT offspring of normoglycaemic (ctrl, NC/HFD, *n*=6/6) and diabetic dams (dbtc, NC/HFD, *n*=6/6), scale bar WGA/COL1/ED1 10/100/20 μm, unpaired Student’s *t* test, mean±SEM, **p≤0.01, ***p≤0.001

For more in-depth characterisation of structural remodelling, immunohistochemistry of cardiac cross-sections was performed. Dbtc offspring, fed an HFD, revealed enlarged cardiomyocytes outlined by significantly increased perimeters in WGA staining with 106.60 ± 4.14 vs 87.94 ± 1.67 μm (Fig. 5a) compared with ctrl, whereas perivascular COL1 content was unaltered with 3.21 ± 0.24 vs 2.79 ± 0.22 (Fig. 5b) in this comparison. The number of CD68-positive cells, as an inflammatory marker, was significantly increased in dbtc offspring compared with ctrl, with 10.27 ± 0.30 vs 6.48 ± 0.48 per fov (Fig. 5c). In contrast, no alterations could be detected in dbtc offspring when being fed NC (WGA 86.05 ± 2.14 vs 81.68 ± 2.84 μm, COL1 3.21 ± 0.24 vs 3.19 ± 0.45 and ED1 7.43 ± 0.53 vs 5.97 ± 0.29 per fov) compared with ctrl.

**Fig. 6:**
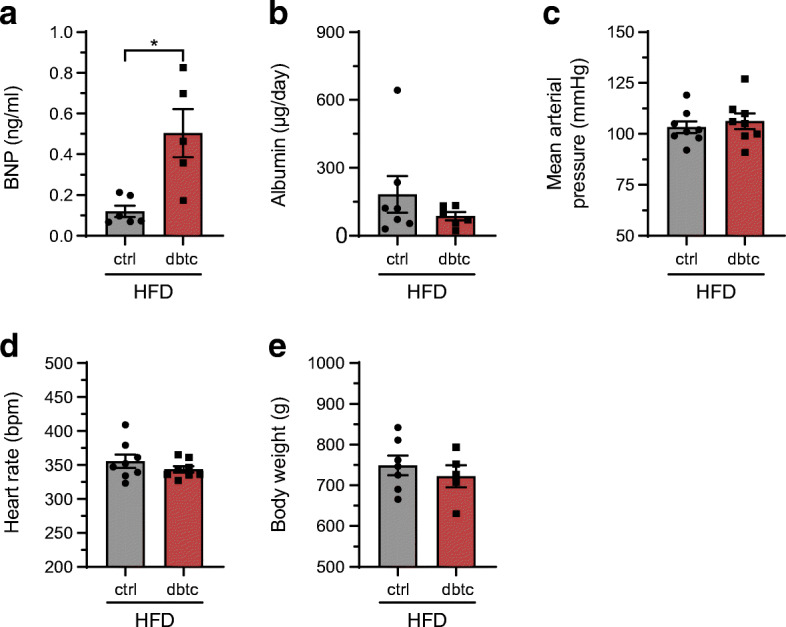
Potential mechanisms driving the cardiovascular phenotype in HFD challenged offspring. Circulatory BNP level was significantly elevated in HFD-fed dbtc offspring compared with ctrl (**a**). Urinary albumin was unaltered in dbtc offspring compared with ctrl (**b**). Likewise, MAP and heart rate were unaltered (**c**, **d**). Body weight was unaltered as well (**e**). Male WT offspring of normoglycaemic (ctrl, HFD; **a**, *n*=6; **b**, **e** *n*=7; **c**, **d** *n*=8) and diabetic dams (dbtc, HFD; **a**, *n*=5; **b**, *n*=6; **c**, **d** *n*=8; **e**, *n*=5), Mann–Whitney *U* test (**a**, **b**) and unpaired Student’s *t* test (**c**–**e**), mean±SEM, *p≤0.05

To investigate whether the pathological remodelling was mediated via classical risk factors, potential drivers leading to the cardiovascular phenotype in the offspring after HFD challenge are shown in Fig. 6. Circulating plasma BNP was significantly increased in dbtc offspring after HFD with 0.50 ± 0.12 vs 0.12 ± 0.03 ng/ml (Fig. 6a) compared with ctrl. Urinary albumin level was unaltered in dbtc with 86.33 ± 18.02 vs 182.43 ± 80.84 μg/day to ctrl offspring after HFD (Fig. 6b). MAP and heart rate were unaltered after HFD challenge in dbtc with 106.25 ± 3.79 vs 103.25 ± 2.91 mmHg and 343.63 ± 4.69 vs 355.50 ± 9.75 beats per minute (bpm) in comparison with ctrl offspring (Fig. 6c,d). Body weight was unaltered with 722.30 vs 748.91 ± 23.74 g (Fig. 6e). In addition, blood glucose, insulin and C-peptide levels and glycaemic control during GTT and ITT were unaltered as shown recently [23]. Systolic BP, diastolic BP and MAP were significantly reduced in NC-fed dbtc offspring in comparison with ctrl and prior to dietary challenge in dbtc offspring compared with ctrl (ESM Table 4).

## Discussion

We have shown in our animal model that diabetic pregnancy leads to cardiac dysfunction after a challenge with HFD in adult offspring. The cardiac phenotype was independent of BP, hyperglycaemia or obesity in the offspring, making the heart a central target for modifications driven by prenatal diabetic condition and postnatal dietary challenge. Myocardial dysfunction in diabetic offspring was detected by STE (reduction in GLS, GLSR, GCS, Tei index) and echocardiographic signs of left ventricular hypertrophy (increased left ventricular posterior wall and RWT) were identified. In alignment with evidence for cardiac dysfunction, an increase in circulating plasma BNP levels was detected. Collected data from immunohistochemistry strengthens the hypothesis of structural remodelling in terms of hypertrophy with enlarged cardiomyocytes and altered proinflammatory status of cardiac tissue in dbtc offspring after HFD. In addition, gene expression in the heart was altered, supporting structural remodelling in terms of hypertrophy (increased *Anp* and *Bnp* expression, which are considered to counterbalance heart failure and cardiac hypertrophy [25]). The remodelling process was accompanied by elevated hypertrophic-cytokine expression in our study (increased *Tnfα*, *Mcp1* expression) inducing an inflammatory environment in the heart tissue. MCP-1 is a chemokine linked to inflammation by attracting and activating monocytes [26]. Elevated *Tnfα* expression is well known in the course of heart failure in humans and mice [27]. On top of the observed hypertrophic and inflammatory cardiac events, a myocardial dysfunction (decreased expression of *Myh6* and *Myh6*/*Myh7* ratio) was detected in our study. MYH6 and MYH7 are motor proteins with MYH6 being considered faster, generating more power than MYH7 [28]. It has been described that MYH6 protein is detectable only in patients without heart failure and undetectable in failing hearts [28], supporting the result of cardiac dysfunction in our study. Our data on cardiac gene expression are generally limited due to the notion that expression patterns underlie structural and functional changes associated with age [29]. In addition, age-onset metabolic changes can be reflected in cardiac gene expression of mice [30]. Recently, Shavlakadze et al performed a gene expression profile of several tissues at multiple time points in rats describing multiple age-related changes throughout life, but unfortunately, the heart has not been investigated [31]. Another trigger of transcriptional changes might be a stressed heart itself. During pathophysiological states such as hypertrophy, hypoxia or diabetes the heart can start a self-protective gene expression programme [32], which appears to be connected to glucose-induced metabolic pathways [32]. We have further shown that a diabetic pregnancy itself is not sufficient to induce cardiac dysfunction in adult male offspring of our transgenic rat model. All together, we provide evidence that a diabetic pregnancy induces cardiac programming, which predisposes adult offspring to cardiac dysfunction after a challenge with an HFD.

One strength of our study is that we assessed cardiac dysfunction by integrating circulating BNP, functional analysis, immunohistochemistry and mRNA expression. BNP is an important marker for cardiac dysfunction [33] and only HFD-fed offspring of diabetic pregnancy displayed increased BNP. We are aware that the dissociated variable of circulating BNP is not sufficient to determine heart failure. However, the progress from normal adaptation to overt heart failure is dynamic and subject to numerous transitions. To specify the value of BNP in the present study, we interpret the circulating BNP increase in connotation with the reduced function in echocardiography as a transition from cardiac maladaptation to diastolic heart failure. STE enables an innovative evaluation of myocardial contractility, both globally and regionally [34]. Thereby it is possible to detect early signs of left ventricular contraction weakness [35]. Being more sensitive than conventional echocardiography, STE can differentiate between pathological and physiological cardiac hypertrophy [36]. In recent years, the possibilities of image acquisition and analysis in animal models have been continuously improved, so that we and others have established STE in rodents as the gold standard for sensitive cardiac phenotyping [35].

Unfortunately, diagnosis, as well as limited treatment options of heart failure with preserved ejection fraction (HFpEF), a representative of cardiac dysfunction, remains unsatisfying at the moment. However, a novel paradigm in this case proposes that myocardial remodelling and dysfunction in HFpEF are the result of non-cardiac comorbidities inducing a proinflammatory status, which in the end results in stiff cardiomyocytes and fibrosis [37]. Non-cardiac comorbidities are highly prevalent in HFpEF, especially hypertension, obesity, diabetes mellitus, chronic obstructive pulmonary disease and anaemia [38]. Here, we describe the prenatal impact of a diabetic pregnancy as a potential non-cardiac event that predisposes to cardiac dysfunction. The novel HFpEF paradigm suggests inclusion of comorbidities and blood markers of inflammation for better diagnosis of HFpEF [37]. Birthweight and pathological pregnancy such as maternal diabetes during pregnancy might be novel candidates worthy of further investigation in this aspect. Offspring birthweight of diabetic and control pregnancies were similar, which may contribute to the unaltered metabolic phenotype in adult offspring of diabetic pregnancy [23]. However, we demonstrate that heart physiology was altered in adult offspring of diabetic pregnancy despite normal birthweight, suggesting the heart as a sensitive target for fetal programming.

Few studies have investigated in detail the effect of a diabetic pregnancy on the heart of the progeny. Miranda et al showed that human fetuses of a diabetic pregnancy exhibit indications of systolic and diastolic dysfunction in the third trimester [39]. Several authors suggested telomere shortening as a potential mechanistic mediator not just of ageing and age-related diseases, but also of fetal programming [40]. Certain circumstances, such as psychosocial stress of the mother during pregnancy [41], conception after assisted reproductive technology [42] or intrauterine stress exposure, are associated with the length of offspring telomeres [43]. If mothers have the metabolic syndrome during pregnancy, it is associated with 14% shorter child telomeres compared with control children [44]. Another stimulating hypothesis includes a diabetic pregnancy-driven hyperglycaemic memory that impacts cardiac stem cells [45]. Gao et al showed that adult offspring of diabetic mice display a greater infarct area and cardiac dysfunction paralleled by increased myocardial apoptosis and oxidative stress after myocardial ischaemia/reperfusion injury [46]. The authors suggested a blunted signalling of cardiac insulin receptor substrate-1/Akt as a mechanism for reduced tolerance to myocardial ischaemia in diabetic offspring [46]. Interestingly, the cardiac phenotype was unaltered between diabetic and normoglycaemic adult offspring at baseline [46], supporting the result of our study that adult offspring on NC do not develop a cardiac phenotype after diabetic pregnancy. This confirms the hypothesis of a second hit during postnatal life (e.g. HFD, myocardial ischaemia/reperfusion [46]) necessary to induce a cardiac phenotype in adolescence after diabetic pregnancy.

We could not determine a potential mechanism linking diabetic pregnancy with offspring cardiac dysfunction, but we can exclude a couple of them within our study. Hypertension is considered a risk factor for cardiac dysfunction but the offspring of diabetic pregnancy in our study did not present higher BP values than the control offspring. However, diabetic offspring in our study might be at a pre-hypertensive stage with already increased peripheral vasoconstriction which might be responsible for the observed cardiac dysfunction. Other authors described an increased vasoconstrictive tone of small vessels [47] and alterations of vascular structure [48] in adult rat offspring after diabetic pregnancy before the development of hypertension. Nehiri et al showed that the hypertension in the offspring of their diabetic pregnancy rat model is salt-sensitive [49], which suggests additional nutritional risk factors for manifestation of hypertension based on fetal programming, which might have been absent in our study. In contrast, hypertension might also be the result, rather than the cause, of cardiac dysfunction. Further studies are needed to elucidate this interaction. Another limitation of our study is the sex-specific aspect, which needs to be further investigated due to rising scientific attention. In the present study, only adult male offspring were tested.

## Supplementary information

ESM (PDF 509 kb)

## Data Availability

The datasets generated during and/or analysed during the current study are available from the corresponding authors on reasonable request.

## Abbreviations

BNP: Brain natriuretic peptide
COL1: Collagen type 1
CTRL: Normoglycaemic dams
ctrl: Offspring of normoglycaemic dams
DBTC: Diabetic dams
dbtc: Offspring of diabetic dams
DOX: Doxycycline
fov: Fields of view
GCS: Global circumferential strain
GCSR: Global circumferential strain rate
GLS: Global longitudinal strain
GLSR: Global longitudinal strain rate
GRS: Global radial strain
GRSR: Global radial strain rate
HFD: High-fat diet
HFpEF: Heart failure with preserved ejection fraction
MAP: Mean arterial blood pressure
MCP-1: Monocyte chemoattractant protein
MYH6: Myosin heavy chain α
MYH7: Myosin heavy chain ß
NC: Normal chow
RWT: Relative wall thickness
shRNA: Small hairpin RNA
STE: Speckle tracking echocardiography
WGA: Wheat germ agglutinin
WT: Wild-type
