# Supplementary material for: Diabetic pregnancy as a novel risk factor for cardiac dysfunction in the offspring—the heart as a target for fetal programming in rats
Source: Diabetologia. 2021 Sep 18;64(12):2829–42. doi: 10.1007/s00125-021-05566-5 (PMC8563640; doi:10.1007/s00125-021-05566-5)
Supplement: Supplementary file 1 — (PDF 509 kb) [file 125_2021_5566_MOESM1_ESM.pdf]

Electronic supplementary material (ESM):

ESM Table 1 Set of primers and probes

Gene		
		Forward primer sequence (5‘-3‘)
		Reverse primer sequence (5‘-3‘)
		Probe sequence (5‘-3‘)
36B4 (Reference)		GCC CAG AGG TGC TGG ACA T
		ATT GCG GAC ACC CTC TAG GA
		ACA GAG CAG GCC CTG CAC ACT CG
Anp Atrial natriuretic peptide		TCT GCC CTC TTG AAA AGC AAA
		CCC GAA GCA GCT TGA CCT T
		CTC TGC TCG CTG GCC CTC GGA
Bnp Brain natriuretic peptide		CAA GCT GCT TTG GGC AGA
		AAA CAA CCT CAG CCC GTC AC
		AGA CCG GAT CGG CGC AGT CAG TCG CTT
Cd68 Cluster of differentiation		AGA CCG GAT CGG AGT CAG TCG CTT
		CAC TTC GGG CCA TGC TTC T
		GGA GGA CCA GGC CAA TGA A
Chrebp Carbohydrate responsive element binding protein		TCA GGG GAT CTC AAC TCC ATT C
		TGC GGA GAT GTG TGT GAT TC
Col1 Collagen type I		AGA GCG GAG AGT ACT GGA
		CTG ACC TGT CTC CAT GTT GCA
		CAA GGC TGC AAC CTG GAT GCC ATC
Ctgf Connective tissue growth factor		AGA GCG GAG AGT ACT GGA
		CTG ACC TGT CTC CAT GTT GCA
		CAA GGC TGC AAC CTG GAT GCC ATC
Et-1 Endothelin 1		AGG GAA AAC CCT GTC CCA AG
		CAC GGG GCT CTG TAG TCA AT
Glut1 Glucose transporter 1		CAC TCA CCA CAC TCT GGT CT
		CAC AAA GGC CAA CAG GTT CA
		CGC TTT GGC AGG CGG AAC TCC A

Glut4<br>Glucose transporter 4		GCC CGA AAG AGT CTA AAG
		TGG ACG CTC TCT TTC CAA CT
		ACA CAT CAG CCC AGC CTG TCA GG
Hif-1<br>Hypoxia inducible factor 1		ATG CTC AGA GGA AGC GAA AAA T
		TGC TGC AGT AAC GTT CCA ATT C
		ACA TGA TGG CTC CCT TTT TCA AGC AGC
Insr<br>Insulin receptor		TGC CCG TCT GGC TAT ACC AT
		TCG AGG ATT TGG CAG ACC TT
Mcp-1<br>Monocyte chemoattractant protein 1		TGC AGT TAA TGC CCC ACT CA
		TCT CCA GCC GAC TCA TTG G
Myh6<br>Myosin heavy chain α		CGG GAG AAC CAG TCC ATC CT
		ACA CGC TTC GTG TTG ACA GTC T
		ATC ACT GAG AAT CCG GAG CGG G
Myh7<br>Myosin heavy chain β		GCC AAG ACA GTT CGG AAT GAT AA
		CCT GTT GCC CCA AAA TGG
		TCC TCC CGA TTT GGG AAA TTC ATT CG
Pecam-1<br>Platelet and endothelial cell adhesion molecule 1		CTC CAT CCT GTC GGG TAA CG
		TTC TTC GTG GAA GGG TCT GC
Tnfα<br>Tumor necrosis factor α		CCT CAC ACT CAG ATC ATC TTC TCA A
		TGC TTG GTG GTT TGC TAC GA
		ACT CGA GTG ACA AGC CCG TAG CCC A
Txnip<br>Thioredoxin interacting protein		AAG GGT CTC AGC AGT GCA AA
		TTC CTG GTC TCA TGA TCA CCA

ESM Table 2 Echocardiographic variables in offspring prior to dietary challenge. Male WT offspring of normoglycaemic (ctrl, *n*=14) and diabetic dams (dbtc, *n*=13), echocardiography assessed at the age of 8 weeks, unpaired Student's *t* test, mean±SEM, *p*-values are given

Echocardiographic variables		ctrl	dbtc	*p*-value
		Mean ±SEM	Mean ±SEM	
Ejection fraction (per cent)		67.86 ±1.34	67.00 ±1.57	0.6819
Fractional shortening (per cent)		38.36 ±1.04	37.14 ±1.12	0.4316
Stroke volume (ml)		301.53 ±11.98	295.37 ±16.61	0.7637
Cardiac output (ml/min)		107.11 ±3.23	94.33 ±5.71	0.0602
Intraventricular relaxation time (ms)		23.65 ±0.33	23.19 ±0.73	0.5766
Intraventricular contraction time (ms)		26.45 ±1.14	26.25 ±1.10	0.8966
Left ventricular mass (g)		847.22 ±12.17	821.69 ±19.83	0.2752
Left ventricle posterior wall (mm)		1.63 ±0.03	1.61 ±0.03	0.5649
Left ventricle inner diameter (mm)		8.45 ±0.09	8.35 ±0.10	0.4724
Intraventricular septum (mm)		1.63 ±0.03	1.61 ±0.03	0.5649
Relative wall thickness		0.39 ±0.01	0.39 ±0.01	0.8425
Tei index		1.06 ±0.05	0.92 ±0.04	0.0627

ESM Table 3 Absolute values of gene expression profile of the heart apex after NC and HFD in reference to housekeeping gene *36B4*. Male WT offspring from normoglycaemic (ctrl, NC *n*=7, HFD *n*=11) and diabetic dams (dbtc, NC *n*=8, HFD *n*=8), qPCR performed at the age of 36 weeks, unpaired Student's *t* test and Mann–Whitney *U* test, normalised mean±SEM

Gene	NC			HFD		
	ctrl Mean ±SEM	dbtc Mean ±SEM	*p*-value	ctrl Mean ±SEM	dbtc Mean ±SEM	*p*-value
36B4	0.52 ±0.05	0.43 ±0.04	0.3671	0.45 ±0.02	0.49 ±0.01	0.3651
Anp	0.92 ±0.35	1.15 ±0.57	0.8665	0.13 ±0.03	1.17 ±0.16	<0.0001
Bnp	0.93 ±0.17	1.07 ±0.37	0.6943	0.68 ±0.08	1.05 ±0.07	0.0051
Col1	1.12 ±0.15	1.07 ±0.10	0.5358	1.09 ±0.07	1.14 ±0.06	0.6339
Ctgf	0.83 ±0.11	0.75 ±0.17	0.6126	0.72 ±0.07	0.68 ±0.07	0.6717
Tnfα	1.24 ±0.13	1.37 ±0.15	0.5015	0.86 ±0.10	1.17 ±0.09	0.0525
CD68	0.91 ±0.23	0.79 ±0.15	0.8665	0.77 ±0.06	0.82 ±0.04	0.5257
Hif1	0.94 ±0.07	0.90 ±0.14	0.6620	0.77 ±0.06	0.82 ±0.08	0.4292
Mcp1	0.84 ±0.07	0.79 ±0.16	0.7973	0.65 ±0.05	1.09 ±0.08	0.0002
Myh6	1.18 ±0.11	1.00 ±0.15	0.3829	1.31 ±0.05	1.04 ±0.06	0.0040
Myh7	1.28 ±0.11	1.41 ±0.18	0.5566	0.71 ±0.06	1.01 ±0.10	0.0165
Pecam1	0.91 ±0.19	1.05 ±0.21	0.6347	0.70 ±0.11	1.23 ±0.10	0.0035
Et-1	1.24 ±0.10	1.02 ±0.18	0.3297	1.23 ±0.14	1.16 ±0.10	0.7019
InsR	1.10 ±0.02	0.96 ±0.09	0.1649	1.03 ±0.03	1.05 ±0.03	0.7441
ChREBP	1.34 ±0.09	1.25 ±0.20	0.6738	1.27 ±0.08	1.33 ±0.12	0.6578
TXNIP	0.60 ±0.08	0.50 ±0.09	0.2319	0.93 ±0.08	0.92 ±0.11	0.9369
Glut1	1.11 ±0.08	1.05 ±0.14	0.7525	0.91 ±0.04	0.85 ±0.05	0.3536
Glut4	1.09 ±0.08	1.01 ±0.12	0.6305	1.06 ±0.06	0.85 ±0.03	0.0135

ESM Table 4 Blood pressure and heart rate of WT offspring prior to and after dietary challenge. Tail-cuff method; Male WT offspring of normoglycaemic (ctrl, prior/NC/HFD, *n*=16/8/8) and diabetic dams (dbtc, prior/NC/HFD, *n*=16/8/8), unpaired Student's *t* test and Mann–Whitney *U* test, mean±SEM, *p*-values are given

Blood pressure measurements by tail-cuff method	Prior challenge			NC			HFD		
	ctrl	dbtc	*p*-value	ctrl	dbtc	*p*-value	ctrl	dbtc	*p*-value
	Mean ±SEM	Mean ±SEM		Mean ±SEM	Mean ±SEM		Mean ±SEM	Mean ±SEM	
Systolic blood pressure (mmHg)	149.13 ±3.49	132.50 ±3.19	0.0014	126.13 ±1.27	116.75 ±3.28	0.0092	127.75 ±3.02	129.75 ±4.25	0.7071
Diastolic blood pressure (mmHg)	107.56 ±3.53	93.19 ±2.46	0.0022	93.25 ±1.90	82.00 ±0.93	0.0002	91.63 ±2.97	94.88 ±3.46	0.4874
Mean blood pressure (mmHg)	121.06 ±3.47	105.94 ±2.68	0.0017	103.75 ±1.70	92.29 ±1.08	0.0001	103.25 ±2.91	106.25 ±3.79	0.5403
Heart rate (bpm)	372.63 ±5.70	359.06 ±8.46	0.1936	347.13 ±5.86	327.75 ±11.81	0.0990	355.50 ±9.75	343.63 ±4.69	0.2908

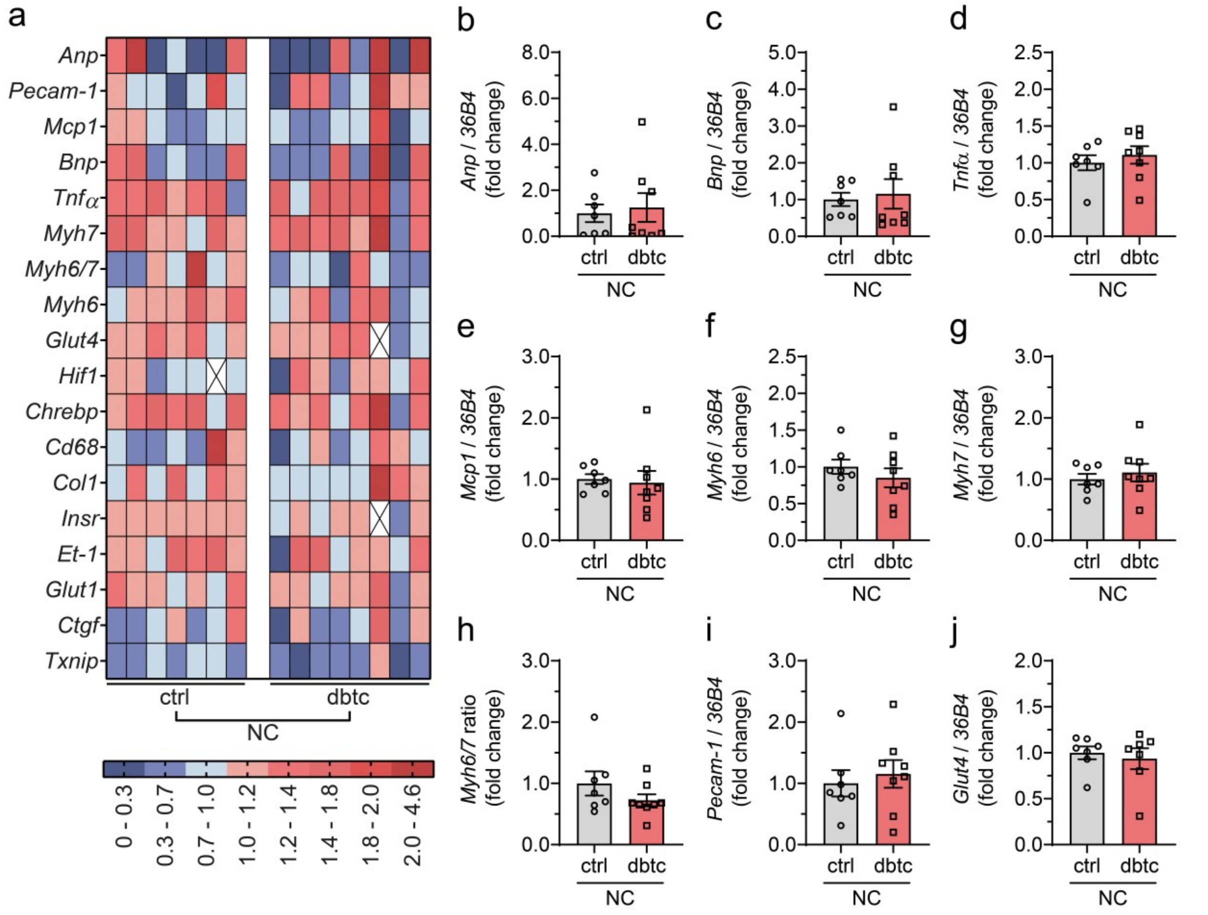

ESM Fig. 1 Cardiac gene expression profile of NC-fed offspring shown as heatmap and selected genes. Gene expression profile of the heart apex after NC challenge illustrated as a heatmap (expression visualised by colour scale) showing absolute values (**a**) and selected genes (**b-j**) in reference to housekeeping gene *36B4*. Dbtc offspring exhibited an overall unaltered gene expression profile compared with ctrl. Offspring from normoglycaemic (ctrl, *n*=7) and diabetic dams (dbtc, *n*=8), qPCR performed at the age of 36 weeks, unpaired Student's *t* test (**d**, **e**, **f**, **g**, **h**, **i**) and Mann–Whitney *U* test (**b**, **c**, **j**), single values (**a**), outlier marked with X (**a**), normalised mean±SEM (**b-j**)
